# Registrars as teachers: a qualitative study exploring the experiences of Australian general practice registrars in teaching roles

**DOI:** 10.1186/s12909-024-06220-5

**Published:** 2024-10-24

**Authors:** Katie Fisher, Irena Patsan, Linda Klein, Alexandria Turner, Dave Runciman, Alison Fielding, Michael Tran, Dimity Pond, Michelle Guppy, Chris Starling, Angelo D’Amore, Andrew Davey, Parker Magin

**Affiliations:** 1 School of Medicine and Public Health, University of Newcastle, University Drive, Callaghan, NSW 2308 Australia; 2 GP Training Research, Royal Australian College of General Practitioners, Level 1, 20 McIntosh Drive, Mayfield West, NSW 2304 Australia; 3 General Practice Clinical Unit, Faculty of Medicine, The University of Queensland, Brisbane, QLD 4006 Australia; 4 School of Clinical Medicine, Discipline of General Practice, Faculty of Medicine and Health, University of New South Wales, High Street and Botany Road, Kensington, Sydney, NSW 2052 Australia; 5 Wicking Dementia Research Education Centre, University of Tasmania, Hobart, TAS 7000 Australia; 6 School of Rural Medicine, University of New England, Armidale, NSW 2351 Australia; 7 GP Training Medical Education, Royal Australian College of General Practitioners, Level 1, 20 McIntosh Drive, Mayfield West, NSW 2304 Australia; 8 School of Rural Health, Monash University, Bairnsdale, VIC 3875 Australia; 9 School of Population Health, Faculty of Medicine and Health, University of New South Wales, High Street and Botany Road, Kensington, Sydney, NSW 2052 Australia

**Keywords:** General practice, Teaching, GP trainees, GP registrars, Medical students, General practice training, Vocational education

## Abstract

**Background:**

GP registrars (specialist vocational trainees in general practice) are interested in teaching, and there are considerable benefits to teaching during training. There are, however, significant barriers for registrars as teachers, including inadequate funding, time pressures, and limited teacher training. Current evidence does not include medical educator (ME) perspectives or compare teaching settings (e.g. university vs. in-practice). Further evidence is needed to inform programs supporting registrar teaching roles.

This project aimed to explore the experiences of Australian GP registrars as teachers in different contexts and from multiple stakeholder perspectives.

**Methods:**

A qualitative study with GP registrars, GP supervisors, MEs, and medical students was conducted. Participants were registrars and new (within 12 months) Fellows with teaching experience during training, supervisors who supervised a registrar in the preceding 12 months, Royal Australian College of General Practitioner MEs, and medical students with experiences of being taught by registrars. Recruitment was open to participants nationwide and sampling was purposive, aiming for a maximum variation sample. Data collection was performed via videoconference and analysed using reflexive thematic analysis.

**Findings:**

Interviews were conducted with 15 registrars, 10 supervisors, and one ME. Two focus groups involved four MEs and five medical students respectively. Registrar participants taught in a variety of settings, including in-practice, universities, hospitals, and at educational workshop days. Three had experience in GP academic posts and one as a registrar ME. There were four major themes. 1) Near-peer teaching by registrars is valuable - both for medical students and registrars. 2) Teaching makes you a better GP - participants noted the transferability of teaching skills to clinical practice. 3) The importance of the teaching context – this was identified as an important determinant for registrars in teaching roles. 4) Registrar teaching strengthens the GP workforce – participants noted that teaching could elevate general practice as a specialty and increase interest in GP training.

**Conclusions:**

Study participants saw teaching as a core skill for GPs, with transferability to their clinical practice. Registrar participants wanted greater promotion and support for teaching opportunities that counted towards attainment of Fellowship. These findings have implications for teaching practices, MEs, universities, and training providers.

**Supplementary Information:**

The online version contains supplementary material available at 10.1186/s12909-024-06220-5.

## Background

General practice registrars (vocational trainees in general practice/family medicine) are interested in teaching [1–3]. However, unlike hospital settings where registrar involvement in teaching is normalised and expected, unique challenges to teaching exist within primary care settings [4]. Chief among them appears to be a cultural attitude towards general practitioner (GP) registrar involvement in teaching, which is seen as a deviation from the norm [4]. An Australian survey found that only 52% of GPs agreed that registrars had the capability to teach in general practice [2]. GP registrar teaching also tends to be ad-hoc and informal, without prior training or protected time [5]. These findings are in contrast to the Royal Australian College of General Practitioners’ (RACGP) curriculum, which includes the core competency “GPs mentor and teach” [6]. Unlike Australia and the United Kingdom, North America has formally adopted ‘residents-as-teachers’ curricula into their vocational training programs, and this has resulted in a high proportion of family medicine trainees being involved in teaching [7, 8]. 

Australian GP registrars are therefore an underutilised teaching resource in the context of an increasing medical student cohort [9] and growing demand for general practice community placements. An increasing mis-match of teaching demand and teaching capacity will be further contributed to by the projected shortfall of 11,392 (28%) full-time GPs in Australia by 2032 [10]. Exposing medical students to high-quality GP placements early in their training can improve perceptions of general practice as a career pathway and increase recruitment to training [11, 12], which is necessary for strengthening the future GP workforce.

However, significant barriers exist to GP registrars’ involvement in teaching, including inadequate funding, time pressures, and limited teacher training [2, 13, 14]. In addition, Australian GP registrars have raised concerns about adequate remuneration, as practice incentive payments (PIP) for teaching are not usually allocated to the registrar [13–15]. Some registrars have also reported that teaching was not a high priority during their training due to competing clinical needs [16]. 

Published literature in this area is incomplete. First, whilst there are studies addressing in-practice GP supervisors’ perspectives of GP registrars as teachers, few include perspectives of external medical educators (MEs). MEs have unique insights due to their involvement in vocational GP training. Further, there are no studies comparing teaching contexts (e.g., in-practice versus university versus hospital settings), with literature focussing on in-practice teaching experiences.

This study aimed to explore the experiences of GP registrars as teachers in different contexts and from multiple stakeholder perspectives.

## Methods

### Study design

This qualitative study used semi-structured interviews and focus groups within a reflexive thematic analysis methodology with an interpretative paradigm [17, 18]. 

### Australian context

In Australia, GP registrars are vocational trainees in general practice/family medicine. On completion of specialty training, GP registrars obtain fellowship with one of the two Australian GP colleges, thereby becoming a GP Fellow. Most Australian registrars will obtain fellowship with the Royal Australian College of General Practitioners (RACGP).

GP registrars train in an apprenticeship-like model, working clinically under the guidance of an experienced GP (supervisor), with a formal education program delivered by regional medical educators (MEs). MEs are GP Fellows with an interest in medical education and relevant education experience in a GP context.

In terms of formal teaching opportunities in training, there are two main roles that registrars can apply for, both of which are salaried. One is the Registrar Medical Educator (RME) role, which is a part-time extended skills role in medical education that is offered by the RACGP. RMEs are eligible to apply for this role once they have completed their first two six-month GP training terms (GPT1 and GPT2). The second is an Australian General Practice Training Academic Post, which is a 12-month part-time role working at an Australian university and involves a combination of research and teaching experience. This term is usually completed alongside part-time clinical work.

Informally, registrars can participate in teaching opportunities within their practices. However, there is no dedicated funding for this teaching unless the registrar’s practice agrees to include this in their paid working hours, or if the practice agrees to allocate the teaching PIP to the registrar. For registrar income, most Australian GP registrars are employed under a contract, where they are paid a base rate of pay, with a ‘top-up’ payment, being the difference between their base rate and a percentage of their billings (with the practice receiving the remaining percentage). The fewer patients a registrar sees, and therefore the less they bill, the lower their take-home pay will be.

### Participants

Registrars, supervisors, MEs, and medical students were invited to express interest in participation via email. Sampling frames included all registrars and supervisors involved in training via RACGP and all MEs employed by the RACGP at the time of recruitment. For medical students, emails were distributed by the following universities: University of Newcastle, University of New England, University of Tasmania, Monash University, and Charles Darwin University. These universities were contacted as the study investigators had affiliations or established contacts for these institutions. Logistics dictated contacting all Australian universities with medical schools to obtain approvals for student recruitment, which was not practicable.

Email invitations were sent to 4,844 registrars, 6,331 supervisors and 325 MEs nationwide. Responses were received from 40 registrars, 48 supervisors, and 7 MEs. The total number of invitations sent to medical students is not known as universities were directly responsible for disseminating invitations to students, however, there were 12 medical student responses received.

Inclusion criteria were:

- GP registrars: registrars and New Fellows (achieved Fellowship within the previous 12 months) with teaching experiences during their GP training.
- GP supervisors: supervisors working in teaching practices where GP registrars have opportunities to teach, and who have supervised at least one registrar within the previous 12 months.
- MEs: all RACGP MEs were eligible to participate.
- Medical students: medical students with experiences of being taught by a GP registrar.

Participants were selected via purposive sampling using a brief questionnaire, with maximum variation sampling considering demographics and teaching experiences and, for registrars, their interest in future teaching. Registrars, supervisors, and medical students were compensated for their time in the form of digital gift cards. MEs were able to participate during their paid working hours.

### Data collection

KF and IP led the data collection and analysis. KF is a female GP and researcher who taught medical students as a registrar, and IP is a female senior research assistant with experience in GP vocational training research and a clinical background in nutrition and dietetics. The rest of the authorship team was comprised of undergraduate/postgraduate MEs, primary care researchers, and GPs. The team’s diversity brought a variety of views and experiences relating to the data. Authors practiced reflexivity, acknowledging their role in the research, and critically appraised their subjective perspective.

Semi-structured interviews and focus groups were performed via videoconference. Interviews were conducted with supervisors and registrars, who were the main stakeholders of this research. Two focus groups, one with MEs and the second with medical students, were performed to provide further context and a deeper understanding of findings from the registrar and supervisor interviews. Focus groups allowed for multiple perspectives to be collected to further enrich the data and enabled project completion within the grant funding period. Informed consent and confidentiality were confirmed prior to interview/focus group commencement. Interview and focus group questions are included in the Supplementary File.

KF and IP led one focus group each while the other took notes. For the two focus groups, the researchers and participants were joined by a senior researcher, LK. Interviews and focus groups were conducted between September 2023 and February 2024. Interview duration ranged from 19 to 60 min, and the focus groups took 73 min and 67 min for MEs and medical students respectively.

KF had taught one of the medical student participants prior to study commencement. KF and IP had working relationships with three ME participants prior to study commencement, through mutual employment at the RACGP.

### Analysis

Interviews and focus groups were audio-recorded, transcribed, and analysed in NVivo qualitative analysis software (version 14.23.1(38)). Memo notes were taken during, and immediately after, data collection. Transcripts were returned to interview participants for respondent validation (but not to focus group participants as deidentified transcripts had potential for participants to alter others’ responses).

Vygotsky’s social constructivist theory was used as a theoretical lens during thematic analysis. This educational theory places emphasis on the influence of the social environment and peer interactions on learning [19]. The transcripts were co-coded inductively by KF and IP, using a combination of semantic and latent coding. KF, IP and LK met regularly to reflect on the interpretation of the data and the theoretical assumptions of Vygotsky’s social constructivist theory. Other members of the authorship team provided input on the findings as the analysis proceeded. KF and IP reflexively iterated the codes and themes were generated by grouping codes. Themes were mapped to produce a framework for the findings.

Thematic saturation was reached for registrars and supervisors, but not for MEs and medical students. However, medical student and ME perspectives provided rich context and explanatory depth for the registrar and supervisor interviews [20]. 

### Ethics

Ethics approval for this project was granted by the RACGP National Research & Evaluation Ethics Committee (NREEC 23–181).

## Findings

Fifteen registrars, 10 supervisors, and one ME (who was unavailable for the focus group) completed interviews. Five medical students and four MEs participated in focus groups. Participant characteristics are presented in Table 1, with teaching experiences for registrar participants summarised in Table 2. Most registrar participants had teaching experiences before commencing GP training and all registrars expressed a desire to teach in the future. Registrar participants taught a variety of learners, including medical students, junior doctors, other registrars, GP Fellows, and allied health practitioners.

**Table 1:** Characteristics of participants

Demographics		Registrars (*n* = 15)	Supervisors (*n* = 10)	Medical educators (*n* = 5)	Medical students (*n* = 5)
Gender	Male	5	4	3	2
	Female	10	6	2	3
Age	< 30 years	5	0	0	5
	30–39 years	8	2	2	0
	40–49 years	2	1	2	0
	50–59 years	0	2	1	0
	60 + years	0	5	0	0
International medical graduate	Yes	2	2	1	
	No	13	8	4	
State/Territory^a^	New South Wales/Australian Capital Territory	4	4	2	4
	Victoria	3	2	1	0
	Queensland	3	1	2	0
	Tasmania	1	2	0	1
	Western Australia	2	0	0	0
	South Australia	2	1	0	0
	Northern Territory	1	0	0	0
Rural placement during training^b^	Yes	11			
	No	4			
Working in a rural practice^b^	Yes		6		
	No		4		
Years of supervisory experience	< 10 years		3		
	10–20 years		2		
	> 20 years		5		
Years in ME role	< 5 years			4	
	5–10 years			0	
	> 10 years			1	

^a^ One registrar had worked in two states during their training

^b^ Classified as Modified Monash Model (MMM) 2–7 (regional, rural or remote area)

**Table 2:** Teaching experiences of GP registrar participants

Teaching experiences	Registrars (*n* = 15)
Teaching in general practice	14
Teaching in university	9
Teaching in hospital	5
RACGP^a^ Academic Post^b^	3
Registrar medical educator^c^	1

^a^ Royal Australian College of General Practitioners

^b^ A 12-month part-time training term involving research and teaching with a local university

^c^ A part-time training term working in medical education with a local GP training team (mentored by local medical educators)

### Theme 1: near-peer teaching by registrars is valuable

Near-peer teaching was defined as registrars teaching medical students and/or prevocational doctors. Participants described the value of this teaching, with benefits for registrars and their near-peer learners.

Medical student participants had overwhelmingly positive learner experiences with registrars. Students described registrars as approachable and more likely to actively involve them in clinical consultations when compared to more senior GPs. Students described teaching from registrars as a “low pressure environment” where they were given permission to make mistakes. Registrars were also passionate about parallel consulting (where the learner sees a patient independently and is joined by the registrar later) and felt this offered the most benefit for medical students on their GP placements.

*“I found the registrars were a lot more willing to get me involved and actually discuss consults and some of their thought processes*,* whereas the senior GPs it was more just sitting and observing. And whenever we had free time*,* they [registrars] were a lot more willing to teach me.” – Medical Student 3*.

*“I think [medical students] really like the near-peer aspect of it. They feel like it’s targeted to them*,* they’re understood as people who are about to become interns because the registrars are only a few years ahead. And I think the students get a vision of what general practice training looks like.” – Supervisor 7*.

Medical students also described registrars as guideline-driven and evidence-based in their practice, which they found helpful for their exam preparation and intern readiness.

*“[Registrar] was very focused on exams and*,* in my case*,* preparing me for internship in giving me tips from GP that would be relevant in my first year of working.” – Medical Student 2*.

Educators (supervisors and MEs) noted benefits of near-peer teaching for registrar confidence and professional identity development. Registrars often spoke about “imposter syndrome” and not feeling experienced enough to teach junior learners, however, educators noted that near-peer teaching could be a validation process that affirmed registrars’ knowledge when compared to more junior learners.

*“There’s often this sense of ‘am I allowed to be teaching?’ You’ve got this imposter syndrome thing going on even though you may have valid clinical experience in the topic and have spent a ridiculous amount of hours reviewing all the literature and evidence for that topic.” – Registrar 3*.

*“‘There’s people in the pipeline who know less than me’ is always a nice thing for registrars. I think that’s quite confidence building.” – Supervisor 7*.

### Theme 2: teaching makes you a better GP

Most registrar participants felt that teaching benefited their clinical practice, keeping them up to date with clinical knowledge, resulting in better exam preparation and patient care. Registrars and educators also noted benefits for reflective practice and improved clinical reasoning. All ME participants felt that teaching was a transferrable and important skill for registrars to learn during their training.

*“I think [teaching] forces you to reflect on your own clinical practice. It prompts that constant self-reflection in terms of ‘how am I practising differently?’ and ‘how am I approaching this patient differently from this student or from this learner?’” – Registrar 7*.

*“I think there’s a lot of skills that we learn in a teaching role that are transferrable to clinical practice. I’m a better clinician for being an educator and vice-versa I think.” – Medical Educator 4*.

Participants noted that improved communication skills were a benefit of teaching experiences, better preparing registrars for teaching their own patients. Several participants noted that the word ‘doctor’ literally translates to ‘teacher’ and defines the job.

*“Every patient encounter is teaching. You’re teaching your patients. I don’t think you can practice in the community as a GP without being a teacher as well and I think learning how to teach…is absolutely vital to being a GP.” – Supervisor 8*.

### Theme 3: the importance of the teaching context

The teaching context was identified as an important determinant of registrars’ teaching experiences. The contexts included teaching practices and the supervisory relationship, universities, and training providers and the ME relationship.

#### Sub-theme: teaching practices and the supervisory relationship

Registrar participants with positive teaching experiences described elements of a rich teaching culture within their practices, including protected teaching time in their appointment book and the role of teaching being shared amongst clinicians in the practice.

*“This practice is pretty good because whenever we’ve got a student*,* we know from four weeks in advance that on this day*,* this particular session*,* the student will be sitting in and our practice manager will book people accordingly*,* leaving a bit more catch-up time.” – Registrar 2*.

Registrars and supervisors noted that remuneration and practice income were significant barriers to teaching in-practice, with income generation being tied only to patient attendances and there being limited funding for teaching. Some registrars reported not receiving the teaching PIP, however, several of the interviewed supervisors felt strongly that this payment should be allocated to registrars if they teach medical students.

*“That payment [PIP]*,* […] you actually don’t see any of that money for having a student sitting in.” – Registrar 12*.

Another common barrier for registrars was lack of opportunity. Some registrar participants noted that their practices had a culture of allocating medical students to senior GPs. Some registrars felt unfounded assumptions were made about whether they wanted to teach and felt that supervisors should be encouraged to find teaching opportunities for registrars in their practices.

*“I think it was just ingrained in that practice that the medical students went with the two principal doctors*,* the way it’d been for many years*,* and it didn’t change.” – Registrar 4*.

*“I think asking registrars if they want to teach. I think people just assume registrars are so busy with the exams and trying to get their head around GP training*,* that they don’t actually get asked.” – Registrar 5*.

In contrast, educator and registrar participants spoke about readiness to teach as an important consideration. Most supervisors felt that registrars in their first 6-month general practice term (GPT-1) would benefit from learning how general practice works before taking on teaching roles. Medical student participants also noted that Fellowship examination periods were not an optimal time for registrars to teach.

*“I really wouldn’t want to give them [GPT-1s] a student straight away until a bit further on. So it just depends on where they’re placing our students*,* the timing*,* and also the confidence of the registrar.” – Supervisor 6*.

*“I can think of one registrar that I was with who was- to be fair they were about to sit their Fellowship exam*,* so they were quite stressed. So that experience wasn’t great*,* I didn’t really get much out of it. It was very much sitting in the corner of the room.” – Medical Student 3*.

One supervisor felt that registrars should have sufficient experience beyond that of their near-peer learners, commenting that registrars could teach medical students but not closer peers.

*“What you really need is to be two pages ahead of the person you’re teaching. Not half a page or one page ahead. So*,* I would trust a registrar with two to three years hospital experience to teach a medical student. I would trust a new Fellow to teach a pre-vocational registrar.” – Supervisor 2*.

#### Sub-theme: university teaching

University-based teaching opportunities varied by region. For three registrar participants, their experiences in university teaching were part of the Academic Post program. For others, it was part-time or casual work performed in addition to clinical duties (for example, teaching case- and problem-based learning tutorials). Opportunities for casual university teaching tended to be informally advertised, with most participants hearing about the work from their colleagues.

Unlike academic post registrars, casual/part-time university teaching rarely counted towards attainment of Fellowship. Some universities had formalised extended skills terms for medical education with RACGP accreditation, but this was not available nationwide. Overall, registrars wanted to see more formal teaching opportunities available to them in training.

*“I don’t think there’s anything like – registrars have specialties*,* don’t they? They do GP anaesthetics. There’s nothing really for registrar teaching as a special skill. Maybe there should be.” – Supervisor 1*.

Rural registrars noted that there were unique logistical challenges for them to get involved in university teaching, namely travel time. Some also commented that academic posts often meant moving to an urban area for a 12-month period, which they felt would be detrimental to their rural communities.

*“Travel for further teaching opportunities is a barrier as well. I’ve got a kid*,* so I’ve been offered to […] do OSCE assessment or travel and help out with nearby clinical schools an hour and 15 minutes away*,* and for me right now*,* with childcare and travel*,* it’s not realistic.”* – Registrar 15.

Registrars who had experience in both in-practice and university settings, such as academic post registrars, noted that they found teaching to be more manageable in an academic setting, as they were able to fully devote their time to teaching activities. These registrars found in-practice teaching more difficult due to the demands of their clinical work.

*“I find it really hard*,* because in clinic I feel I’m doing the learning. I feel I don’t have any extra energy in addition to what I’m doing clinically to dedicate to the student[…]. So I find it really challenging having them in-clinic*,* compared to [university]*,* it’s a very different environment*,* 100% of my focus is teaching and I find it easier.” – Registrar 11*.

#### Sub-theme: training providers and the ME relationship

MEs and the RACGP (being the main training provider in Australia) have an opportunity to support registrars in teaching roles. Firstly, registrars wanted their teaching experiences to count towards Fellowship and be formally recognised.

*“I think a lot of registrars have an interest in teaching but it’s the opportunity to do it within training as well. Because I know some registrars tried to have a day a week in a medical education position but it wasn’t recognised in a training capacity.” – Registrar 12*.

Secondly, some registrars expressed a need for teaching skills training from the RACGP. This sentiment was more salient amongst registrars participating in teaching in-practice, compared to those in academic teaching roles who reported greater access to teacher training (for example, via Academic Post program or university departments). However, not all registrars identified a need for formal training, with many commenting that they learned by observing their supervisor or via experiential learning.

Thirdly, registrars were divided on how well they felt teaching opportunities, like academic posts and registrar ME roles, were promoted by the RACGP and their MEs. Some felt there was ample promotion whereas others reported seeking their own information or hearing about opportunities from peers.

*“I think it’s honestly word of mouth*,* otherwise it’s [Academic Post program] not really widely distributed through RACGP or [regional training organisation] like what it was before.” – Registrar 13*.

Specific to the RACGP GP training curriculum, participants were divided on whether teaching should be compulsory in training, despite most participants commenting that teaching was a valuable skill. Many participants identified that not all registrars would be suited to teaching, particularly if they were struggling with their own learning needs.

*“Teaching junior colleagues can’t be an obligation on registrars who aren’t ready for it*,* or who aren’t suited to it.” – Supervisor 2*.

*“If you don’t start with [teaching] when you’re training*,* how are you supposed to continue that as a consultant?” – Registrar 3*.

Regarding RACGP accredited extended skills terms, registrars and educators felt positively about a medical education post for interested registrars. Some participants noted that registrar ME positions were already available and filled this need, however, there are limited positions nationwide (generally one per training region). Contrary to other educator views, one supervisor felt that a medical education post would reduce registrars’ clinical exposure during a relatively short two-year training program (when compared to hospital-based training programs).

*“Anything that takes registrars out of the consulting room better be damned valuable if it’s going to be worthwhile for the profession and for the community*,* and I doubt very much that registrars doing teaching is.” – Supervisor 2*.

However, registrars and educators noted that teaching did not necessarily have to detract from clinical exposure, with the opportunity to combine teaching with clinical medicine, for example, parallel consulting with junior learners.

*“I don’t think it’s a significant detractor to have that time spent on education. And given all the other valuable things you’ll take from learning how to teach and communication benefits*,* learning how to update yourself*,* I actually think would be a really valuable experience.” – Registrar 10*.

### Theme 4: registrar teaching strengthens the GP workforce

#### Sub-theme: teaching improves the reputation of general practice

Participants noted that registrars teaching junior learners had the potential to improve the perception of general practice and increase medical student interest in a GP career. Participants contrasted general practice and hospital environments, noting that teaching could elevate general practice to align with other specialties.

*“I think teaching is a really big part of other training specialties in hospitals and I think it elevates the practice*,* elevates the specialty […]. Yeah I think it’s elevating to teach.” – Registrar 9*.

Participants also noted that junior learners would have a better appreciation of general practice, even if they did not go on to pursue a general practice career.

*“But we might be just helping [medical students] to see the value of general practice. And I think that works both ways. The medical student might come back as a GP*,* but they might also*,* from the hospital*,* understand general practice a bit better.” – Supervisor 10*.

To contrast these views, medical student participants did not note any differences in the career advice obtained from GP registrars compared to senior GPs, finding both perspectives useful for their own decision-making. Medical students also commented that they would prefer work-based experiences to support their career decisions, such as general practice placements in residency, which are not widely available.

#### Sub-theme: a teaching role improves career sustainability

Participants found that teaching roles improved job satisfaction and made their GP careers more sustainable in the long-term. Some registrars noted that teaching medical students in-practice allowed them to slow the pace of their clinical day and reduce social isolation. Many registrars spoke about the risk of burnout with full-time clinical work and found that splitting their week between clinical and educational work was favorable. ME participants echoed these views, noting the role their own education careers played in reducing burnout.

*“The other thing I liked about [teaching] was*,* while I love clinical work*,* it reduces the clinical load because I’m happy to work full-time*,* but full-time clinical work in GP land is tiring.” – Registrar 12*.

*“Educating’s fun. I mean*,* we’re biased*,* we’re the ME group. Another thing it can do is really prolong careers and with me it prevents burnout.” – Medical Educator 3*.

Educator participants also noted that registrar teaching roles were an important part of succession planning, by training up the next generation of GP medical education workforce.

*“Benefits in terms of their confidence and I think when they Fellow they’re much more likely to be keen to take on students*,* registrars and interns and be a supervisor” – Supervisor 3*.

## Discussion

### Summary of the main findings

Teaching is a core competency within the RACGP curriculum [6], and participants recognised the importance of this skill. However, teaching is not currently a formal requirement of the RACGP training program, possibly reflecting existing cultural attitudes towards GP registrars as teachers. Participants were divided on whether teaching should be mandatory during training, noting the competing priorities of the training program and considerations around readiness to teach. Registrars and educators noted benefits and challenges that were consistent with previous research [21], however, positives far outweighed the negatives. A novel finding in this study was the transferability of teaching skills to clinical practice, with participants noting the benefits in teaching their patients and providing more evidence-based care. Our findings also suggest that teaching has the potential to strengthen the GP workforce, by increasing interest in GP training and providing career sustainability.

The training context and their relevant stakeholders play an important role in supporting registrars to adopt teaching roles. There exists an opportunity for supervisors, MEs, universities, and training providers to promote teaching pathways for registrars. Most participants felt that increased teaching in general practice had the potential to elevate the specialty and provide greater parity with hospital-based training programs.

### Consideration of findings with our theoretical lens

Our findings on the utility of near-peer teaching can be seen through the lens of existing educational theory, including Vygotsky’s social constructivist theory [16, 19, 22]. Thampy et al. saw the benefits of the ‘near-peer tutor’ having social and cognitive congruence with the learner, allowing registrars to more readily identify their learners’ zone of proximal development (ZPD), which, in Vygotsky’s model, represents the difference between a learner’s current knowledge level and the potential level they can achieve through social interaction with a more experienced tutor [16, 19]. This was supported by medical student participants, who noted social congruence with registrars, who were described as more approachable, and cognitive congruence, with registrars being more examination- and internship-oriented. Students also described teaching with registrars as a “low pressure environment”, which allowed them to make mistakes and thereby develop within their ZPD. These findings are congruent with existing literature [15, 16, 23–25]. 

Our findings also suggest that near-peer teaching assists with registrars’ professional identity development, both as teachers and clinicians. Educator participants commented on the role that near-peer teaching played in improving registrars’ confidence levels and consolidating their clinical knowledge. This is consistent with undergraduate medical education research [22], which describes the near-peer tutors’ professionalisation being facilitated within a social-learning environment, including role-modelling behaviour to tutees and via experiential learning.

While Vygotsky’s social constructivist theory is useful for considering our findings on the value of near-peer teaching, it is unable to permit exploration of the tension between clinical and educational roles within general practice. Some of our participants could appreciate the complementary nature of these roles, and the benefits of such coupling. There were even suggestions that better integration of near-peer teaching into the vocational training clinical setting could contribute to to the sustainability of general practice in Australia.

### Teaching extended skills term

It has been suggested that an accredited teaching career pathway, or teaching fellowship, could assist in increasing uptake of registrars in teaching roles [15, 26]. A teaching fellowship would allow senior GP registrars to work in dedicated teaching positions, complete teaching skills training, and facilitate capacity-building for medical education in general practice. This concept was received positively by registrars, although there were some concerns about reduced clinical exposure in the relatively short two-year training program.

Our findings demonstrated that this type of accredited extended skills term was available at some Australian universities, however, this was not widely available nationwide. The limited number of accredited positions may reflect the existing cultural attitudes towards GP registrars as teachers. Increasing the availability of these positions could help to normalise teaching in training, similar to North America’s ‘resident-as-teacher’ programs [7]. 

### Implications for stakeholders and further research

Our findings have implications for relevant stakeholders, including teaching practices and supervisors, medical educators, universities, and the RACGP as the major training provider in Australia. Increasing the number of formal teaching pathways in training will require funding and RACGP accreditation. In addition, registrars would benefit from the provision of formal teacher training before commencing teaching roles and the RACGP is well placed to deliver this training.

Future pilot programs are needed to develop sustainable and practical models of teacher training within Australian general practices, which is where most teaching will occur due to limited university appointments. Formal and recognised teacher training is also necessary to change the perception of registrars as teachers within general practice, and to support the interdependency of clinical and educational roles in primary care more broadly. Future research is needed to explore the feasibility and scalability of a registrar teaching program nationwide.

The RACGP could consider greater promotion of teaching pathways and look to expand registrar ME positions nationwide. Teaching practices and GP supervisors can also support registrars in their teaching endeavours by considering ways to involve them in teaching opportunities with the practice (for example, evidence-based journal clubs and practice education meetings), allowing protected ‘catch-up’ time in their appointment books for teaching, and remunerating the registrar where appropriate. Lastly, university-affiliated teaching opportunities would be better incentivised if they were counted towards attainment of GP Fellowship via RACGP accreditation.

### Strengths and limitations

This project recruited participants from around Australia, using maximum variation sampling, which improves transferability of our findings, and allows for comparisons between important stakeholder groups. Reflexivity and investigator collaboration were performed regularly, including memo notes and regular discussion between IP and KF during co-coding and thematic analysis. Finally, credibility was increased by employing respondent validation, with participants verifying their transcripts and editing to ensure accuracy.

Medical student responses may have been influenced by a perceived power imbalance during the focus group. Students may have been unwilling to share negative experiences because of social desirability bias. In addition, the majority of students were from one region within NSW, which could reduce the transferability of these findings.

Lastly, all registrars who expressed an interest in participating in the study were interested in teaching in the future. Experiences and attitudes of registrars without an ongoing intent to teach are absent from the data.

## Conclusion

Study participants saw teaching as a core skill for GPs, with transferability to their clinical practice. Near-peer teaching was seen as valuable for medical students, through social and cognitive congruence, and for registrars, in the development of their professional identity. Registrar participants wanted greater support for teaching opportunities that counted towards attainment of Fellowship. These findings have implications for teaching practices, MEs, universities, and training providers.

## Supplementary Information

Supplementary Material 1.

## Data Availability

The data that were used in this study cannot be publicly shared due to ethical and privacy concerns. Informed consent, in line with the approving ethics committee, only allows the use of de-identified extracts within research reporting and writing, to maintain the privacy of participants.

## Abbreviations

CV: curriculum vitae
GP: general practitioner
GPT-1: general practice term 1
GPT-2: general practice term 2
ME: medical educator
OSCE: objective structured clinical examination
PIP: practice incentive payment
RACGP: Royal Australian College of General Practitioners
RME: registrar medical educator
ZPD: zone of proximal development
