# Supplementary material for: Registrars as teachers: a qualitative study exploring the experiences of Australian general practice registrars in teaching roles
Source: BMC Med Educ. 2024 Oct 24;24:1203. doi: 10.1186/s12909-024-06220-5 (PMC11515485; doi:10.1186/s12909-024-06220-5)
Supplement: Supplementary file 1 — Supplementary Material 1. [file 12909_2024_6220_MOESM1_ESM.docx]

**Semi-structured interview guide for GP registrars**

1. Can you please tell me about your experiences of teaching as a GP registrar? This could include teaching medical students in-practice, medical students at university, other registrars/peers in practice, or GP Fellows.
2. Do you have experience of working in a formal teaching role, for example, as a GP Academic Registrar, as a Registrar Medical Educator, or as a university lecturer? Can you tell me about those experiences?
3. How have these experiences influenced your desire to teach in the future?
4. What are your views on whether GP registrars should teach routinely as part of their training?
5. Do you have experiences of being taught by a GP registrar in the past? How have those experiences influenced your views towards GP registrars as teachers?
6. Can you share your experiences of teaching to different learner levels, for example, to medical students, other GP registrars and to GP Fellows?
7. In your experience, what were the benefits of teaching as a GP registrar?
8. In your experience, what were the challenges of teaching as a GP registrar?
9. In your experience, what are the barriers for GP registrars in adopting teaching roles?
10. In your experience, what are the facilitators for GP registrars in adopting teaching roles?
11. Do you have experiences of teaching outside of the in-practice setting? For example, teaching in a university or teaching in a Registrar Medical Educator role? If so, how did these teaching experiences differ?
12. What are your thoughts on dedicated teaching positions for registrars as part of their Extended Skills? For example, spending 6-months working in various teaching roles across different settings and learner levels?

**Semi-structured interview guide for GP supervisors**

1. Can you please tell me about your experiences of supervising GP registrars in teaching roles?
2. Did these experiences influence a registrar’s decision to become a teacher in the future? Can you think of any examples?
3. Thinking back to when you were a GP registrar, did you have any opportunities to teach? Can you tell me about those experiences?
4. What are your views on whether GP registrars should teach routinely as part of their training?
5. Thinking about registrars you have supervised, to what extent have they taught across different learner levels, for example, medical students, other GP registrars in the practice, GP Fellows? If so, how do you think these experiences differed?
6. In your experience, how does teaching to different learner levels impact registrar learning and development?
7. In your experience, what are the benefits for GP registrars in teaching during their training?
8. In your experience, what are the challenges for GP registrars in teaching during their training?
9. In your experience, what are the barriers for GP registrars in adopting teaching roles?
10. In your experience, what are the facilitators for GP registrars in adopting teaching roles?

**Semi-structured focus group guide for Medical Educators**

1. Can you please tell me about your experiences of GP registrars in teaching roles?
2. Did these experiences influence a registrar’s decision to become a teacher in the future, for example, as a GP supervisor or Medical Educator or GP Academic? Can you think of any examples?
3. What are your views on whether GP registrars should teach routinely as part of their training?
4. Thinking about the registrars you have educated, what are the benefits or challenges in registrars teaching across different learner levels; that is, medical students versus GP registrars or GP Fellows in their practice?
5. In your experience, what are the benefits for GP registrars in teaching during their training?
6. In your experience, what are the challenges for GP registrars in teaching during their training?
7. In your experience, what are the barriers for GP registrars in adopting teaching roles?
8. In your experience, what are the facilitators for GP registrars in adopting teaching roles?
9. To what extent are you aware of registrars teaching in different settings? For example, teaching in-practice versus teaching in a university or teaching in a Registrar Medical Educator role? If so, how do you think these teaching experiences differed?
10. What are your thoughts on dedicated teaching positions for registrars as part of their Extended Skills? For example, spending 6-months working in various teaching roles across different settings and learner levels?

**Semi-structured focus group guide for medical students**

1. Can you please tell me about your experiences of being taught by GP registrars?
2. To what extent, if at all, has being taught by a GP registrar affected your perspective on pursuing a career in general practice?
3. In your experience, what are the benefits of being taught by a GP registrar?
4. In your experience, what are the challenges of being taught by a GP registrar?
5. How do these benefits and challenges compare to being taught by a Fellowed GP? (i.e. a GP who has completed their Fellowship training).
6. Do you have experiences of being taught by GP registrars outside of general practice placements, for example, in a university setting (such as lectures or tutorials) or hospital setting? If so, can you please tell me about those experiences?
